# Multiple and frequent tobacco product use by sexual minority youth in the United States: Results from the 2023 National Youth Tobacco Survey

**DOI:** 10.1016/j.pmedr.2025.103069

**Published:** 2025-04-10

**Authors:** Juhan Lee, Rebecca J. Evans-Polce, Maria A. Parker

**Affiliations:** a Department of Epidemiology and Population Health, Albert Einstein College of Medicine, Bronx, NY, United States of America; b Center for the Study of Drugs, Alcohol, Smoking and Health, School of Nursing, University of Michigan, Ann Arbor, MI, United States of America; c Department of Epidemiology and Biostatistics, Indiana University, Bloomington, IN, United States of America

## Abstract

**Objective:**

Understanding tobacco product use among sexual minority youth is important due to the exposure to nicotine and toxicants, which worsens tobacco-related health disparities. We used recent US national data to characterize tobacco product use by sexual identity.

**Methods:**

Data were drawn from the 2023 National Youth Tobacco Survey (NYTS), a nationally representative survey of US middle and high school students (*N* = 20,503). Sexual identity categories included “heterosexual”, “gay/lesbian”, “bisexual/pansexual/queer”, “asexual”, “questioning”, “I do not know what this question means”, “something else”, and “decline to answer.” Outcomes were past-30-day (1) multiple tobacco product use (number of products) and (2) frequent tobacco product use (≥20 days). Weighted Poisson regression models examined associations between sexual identity and tobacco use, adjusting for covariates.

**Results:**

In 2023, there was more tobacco product use (adjusted prevalence ratio[aPR] = 2.35, 95 % CI = 1.15, 4.79) and more frequent tobacco product use among gay/lesbian (aPR = 2.89, 95 % CI = 1.42, 5.87) and asexual youths (aPR = 2.92, 95 % CI = 1.41, 6.04) (vs. heterosexual youth) in the past 30 days. Compared to heterosexual youth, gay/lesbian and asexual youth were more likely to report combustible tobacco use, while bisexual, pansexual, queer, and questioning youth were more likely to use non-combustible products, such as e-cigarettes and nicotine pouches.

**Conclusions:**

This study observed more multiple and frequent tobacco product use among US sexual minority youth than heterosexual youth. These disparities by sexual identity emphasize the need for targeted tobacco prevention and cessation programs. Public health interventions should address unique stressors and risk factors contributing to tobacco use among sexual minority youth.

## 1 Introduction

The use of multiple tobacco products (i.e., currently using two or more tobacco products) among youth is a significant public health concern due to the potential exposure to high levels of nicotine and toxicants (United States Department of Health and Human Services, 2012). Multiple tobacco product use is related to increased risk of nicotine dependence, difficulty in quitting each tobacco product, and other substance use (US Department of Health and Human Services, 2012). In 2024, 3.0 % of middle and high school students in the United States (US; representing 840,000 students) used two or more tobacco products in the past 30 days (Jamal et al., 2023). Understanding the frequent tobacco product use (i.e., ≥20 days in the past 30 days) (Birdsey et al., 2023; Wang et al., 2019) is also important since frequent tobacco use could be more harmful than the simple cumulative effect of tobacco products used (e.g., one tobacco product frequently vs. two products rarely).

Previous studies have observed a greater number of tobacco products used by sexual minority youth compared to heterosexual youth (Creamer et al., 2019; Hart et al., 2020; Tabaac et al., 2021). For example, past-30-day use of two or more tobacco products was reported by 10.4 % of sexual minority youth in 2019 compared to 7.8 % of heterosexual youth (Creamer et al., 2019). Sexual minority youth are also more likely to report frequent tobacco product use than heterosexual youth (Azagba and Shan, 2021; Watson et al., 2018), which might increase risk of nicotine and toxicant exposure. Higher levels of tobacco use by sexual minority youth might be driven in part by coping with stress from experienced and perceived discrimination or sexual identity concealment (Meyer, 2003). Additionally, it may be impacted by targeted tobacco industry marketing, such as sexual minority symbols (e.g., rainbow flags) in advertising in venues frequented by sexual minority individuals (Blosnich et al., 2013).

There is still a knowledge gap in multiple tobacco product use among sexual minority youth in recent years at the population level. The tobacco product market landscape (e.g., nicotine pouches gaining popularity in recent years) and tobacco regulatory policies are rapidly changing (Robichaud et al., 2020). For example, the sales of nicotine pouches (i.e., “small, dissolvable, flavored pouches containing nicotine derived from tobacco that users place in the mouth between the lip and gum” (Park-Lee et al., 2024)) and other oral nicotine products (e.g., nicotine lozenges and tablets) have increased in recent years, and they were the second most used tobacco products among US youth in 2024 (Park-Lee et al., 2024).

Understanding multiple tobacco product use by subgroups of sexual minority groups such as asexual individuals is also important. New sexual identities are constantly evolving, and new concepts and terminologies (e.g., asexual, plurisexual) are appearing (UCLA Williams Institute, n.d.). Younger people (youth and young adults) are more likely to identify themselves as gender diverse and those numbers are increasing in recent years. (UCLA Williams Institute, n.d.) Furthermore, characterizing tobacco products used and their combinations by sexual identity is important to inform efforts to address multiple tobacco product use among sexual minority youth.

Therefore, an updated investigation that includes newer tobacco products and more sexual identity options is needed to understand multiple tobacco product use by sexual minority youth. This study aimed to use the most recent national dataset to examine multiple tobacco product use and frequent tobacco product use by sexual identity among youth. We hypothesized that sexual minority youth would use more tobacco products than heterosexual youth. This study also characterized the most used tobacco product by sexual identity.

## 2 Methods

### 2.1 Dataset and analytic sample

This study used the 2023 National Youth Tobacco Survey (NYTS), which is the most recently released, publicly available NYTS data (*N* = 20,503). The NYTS is an annual school-based cross-sectional survey collected by the Centers for Disease Control and Prevention that uses stratified, three-stage cluster sampling to represent US middle and high school students (6–12 grades in public and private schools). Randomly selected schools, classrooms and students are recruited to complete an online questionnaire related to tobacco product use. Using sampling weights and accounting for the complex sampling design, the responses in the NYTS represent US middle and high school students. Detailed sampling design and data collection procedures are described elsewhere (Centers for Disease Control and Prevention (CDC), 2020).

The 2023 NYTS assessed updated sexual identity questions to accommodate changing terminology (e.g., pansexual, asexual) and assessed questions about nicotine pouches and other oral nicotine product use (e.g., lozenges, discs, tablets, gums, dissolvable tobacco products, and other products), which have gained popularity in recent years (Park-Lee et al., 2024).

### 2.2 Measures

Youth self-reported use of cigarettes, cigars, smokeless tobacco/snus, e-cigarettes, hookah, roll-your-own cigarettes, pipe, bidis, heated tobacco, nicotine pouches (e.g., Zyn, On!), and other oral nicotine products (e.g., Velo Nicotine Lozenges, Rogue Lozenges, Rogue Tablets). The first outcome was multiple tobacco product use in the past 30 days. We summed the number of tobacco products used in the past 30 days to create a count variable ranging from 0 to 11. The second outcome, frequent tobacco product use (Birdsey et al., 2023), was assessed for each product (0–19 days, 20–30 days) (Birdsey et al., 2023; Wang et al., 2019); we summed the number of tobacco products with frequent use ranging from 0 to 11.

The main independent variable was self-reported sexual identity (Bates et al., 2022), categorized as “heterosexual or straight”, “gay or lesbian”, “bisexual, pansexual, or queer”, “asexual”, “I am not sure or I am questioning” [“questioning”], “I do not know what this question means”, “something else not included here”, and “decline to answer.”

Covariates were selected based on previous literature related to tobacco use among youth (US Department of Health and Human Services, 2012). The covariates included sex assigned at birth (male, female), school level (middle school, high school), race (non-White, White), ethnicity (non-Hispanic, Hispanic), gender identity (cisgender man/woman, nonbinary/genderfluid/or genderqueer, “something else”, “I'm not sure or I am questioning”, “decline to answer”), family tobacco use (none, any), and peer tobacco use (none, any).

### 2.3 Statistical Analyses

First, we described sample characteristics by sexual identity. We then conducted adjusted Poisson regression models on multiple tobacco product use and frequent tobacco product use in the past 30 days as a function of sexual identity, adjusting for covariates. We set alpha = 0.025 (two-tailed) as a statistical significance cut-off for multiple comparison adjustment (0.05/2 models). We also identified the most used tobacco products by sexual identity among youth respondents who had used any tobacco use in the past 30 days.

Next, we conducted two sensitivity analyses. Due to potential overdispersion in the Poisson regression models, negative binomial regression models were conducted (**Supplemental Table 1**). Further, due to potential excess zeros in the number of tobacco products used among overall youth respondents, we also ran zero-inflated Poisson regression models (**Supplemental Table 1**).

We also ran three supplemental analyses. First, we examined associations among the subset of youth who had any past-30-day tobacco product use (*n* = 1977) to examine multiple tobacco product use (**Supplemental Table 2**). Second, since the use of more combustible tobacco products might be more harmful and exposed to more nicotine and toxicants than more non-combustible tobacco products (*National Academies of sciences engineering and medicine*, 2018), we ran separate models for the number of combustible tobacco products (ranging from 0 to 6) (**Supplemental Table 2**) and the number of non-combustible tobacco products (ranging from 0 to 5) (**Supplemental Table 2**) used in the past 30 days.

We used a complete-case approach for analyses and followed the STrengthening the Reporting of OBservational studies in Epidemiology guidelines. Sampling weights and complex survey design of 2023 NYTS were incorporated for population-level estimation. This secondary data analysis of publicly available de-identified national data was determined exempt by the Institutional Review Board. The STATA 18.0 was used for all analyses.

## 3 Results

In 2023, 2.9 % (weighted) of middle and high school students in the United States identified themselves as gay/lesbian, 9.3 % as bisexual, pansexual or queer, 1.2 % as asexual, 3.3 % reported “I am not sure or I am questioning”, 3.1 % reported “I don't know what this question means”, 3.4 % reported something else, and 13.5 % reported “decline to answer.” The prevalence of any tobacco use in the past 30 days was 7.8 % for heterosexual, 16.8 % for gay/lesbian, 16.1 % for bisexual, pansexual or queer, 15.0 % for asexual, 11.9 % for “I'm not sure or I am questioning” group, 7.2 % for “I don't know what this question means” group, 11.2 % for something else, and 7.4 % for the “decline to answer” group. Among youth who used any tobacco in the past 30 days, the weighted mean of tobacco products used was 2.9 among gay/lesbian youth, followed by 2.5 among asexual youth and 2.4 among something else youth. The weighted mean of frequent tobacco product use was 1.3 among asexual youth, 1.1 among something else youth, and 1.0 among gay/lesbian youth (Table 1). The most common tobacco use pattern was “exclusive e-cigarette use” across all sexual identities (data not shown in a table).

**Table 1:** Sample characteristics by sexual identity among total youth respondents in the United States from 2023 National Youth Tobacco Survey (*N* = 20,503; data was collected in 2023).

	Total	Straight or heterosexual	Gay or lesbian	Bisexual, pansexual, or queer	Asexual	I am not sure or I am questioning	I don't know what this question means	Something else	Decline to answer
	20,503 (100)	11,132 (63.3)	493 (2.9)	1602 (9.3)	248 (1.2)	561 (3.3)	631 (3.1)	682 (3.4)	2520 (13.5)
Multiple tobacco product use ^a^									
Weighted mean (SD) (range: 1–11)	1.9 (1.6)	1.7 (1.3)	2.9 (2.0)	1.7 (1.6)	2.5 (2.6)	2.4 (1.9)	2.0 (1.8)	2.4 (2.2)	1.8 (1.4)
Frequent tobacco product use ^a^									
Weighted mean (SD) (range: 1–11)	0.5 (1.1)	0.4 (1.0)	1.0 (1.1)	0.5 (1.1)	1.3 (2.4)	0.3 (0.6)	0.5 (1.1)	1.1 (1.7)	0.5 (1.2)
Sex									
Female	10,015 (49.0)	4933 (43.7)	301 (65.1)	1240 (78.8)	146 (54.1)	373 (63.4)	300 (46.9)	291 (41.6)	1251 (52.2)
Male	10,345 (51.0)	6188 (56.3)	185 (34.9)	340 (21.2)	100 (45.9)	183 (36.6)	328 (53.1)	383 (58.4)	1246 (47.8)
School level									
Middle school	9541 (40.8)	4870 (35.9)	201 (30.5)	616 (34.2)	108 (35.1)	301 (46.9)	418 (66.3)	375 (46.7)	1340 (49.7)
High school	10,849 (59.2)	6248 (64.1)	289 (69.5)	983 (65.8)	139 (64.9)	258 (53.1)	209 (33.7)	301 (53.3)	1168 (50.3)
Race									
White	11,641 (62.1)	6691 (65.7)	298 (64.6)	1009 (68.3)	145 (64.7)	308 (60.5)	313 (51.2)	392 (62.6)	1262 (57.2)
Non-White	8862 (37.9)	4441 (34.3)	195 (35.4)	593 (31.7)	103 (35.3)	253 (39.5)	318 (48.8)	290 (37.4)	1258 (42.8)
Ethnicity									
Non-Hispanic	14,174 (69.4)	8056 (72.9)	342 (71.5)	1110 (72.1)	176 (65.2)	405 (68.8)	419 (60.0)	485 (67.4)	1706 (67.8)
Hispanic	6329 (30.6)	3076 (27.1)	151 (28.5)	492 (27.9)	72 (34.8)	156 (31.2)	212 (40.0)	197 (32.6)	814 (32.2)
Family tobacco use									
None	12,337 (58.6)	7553 (66.4)	265 (52.6)	804 (49.3)	122 (46.3)	358 (57.4)	477 (76.1)	424 (61.0)	1848 (73.6)
Any	8166 (41.4)	3579 (33.6)	228 (47.4)	798 (50.7)	126 (53.7)	203 (42.6)	154 (23.9)	258 (39.0)	672 (26.4)
Peer tobacco use									
None	2800 (13.3)	1425 (11.2)	52 (9.2)	115 (7.3)	34 (9.5)	96 (15.5)	185 (30.2)	89 (13.0)	572 (23.2)
Any	16,325 (86.7)	9658 (88.8)	438 (90.8)	1473 (92.7)	212 (90.5)	461 (84.5)	437 (69.8)	581 (87.0)	1914 (76.7)
Gender identity									
Cisgender	16,199 (89.3)	10,960 (98.8)	348 (70.6)	1159 (73.2)	157 (67.0)	451 (77.5)	561 (88.0)	493 (65.8)	1787 (70.4)
Nonbinary, genderfluid, or genderqueer	518 (2.9)	17 (0.2)	73 (14.8)	255 (14.7)	58 (21.1)	41 (11.3)	4 (0.7)	46 (8.8)	21 (0.7)
Something else	320 (1.9)	67 (0.6)	27 (6.6)	53 (2.6)	13 (5.7)	10 (3.2)	7 (1.2)	115 (21.6)	21 (0.5)
I'm not sure or I am questioning	227 (1.3)	11(0.0)	24 (5.3)	85 (6.9)	14 (5.1)	37 (6.1)	27 (4.7)	14 (1.9)	11 (0.2)
Decline to answer	814 (4.6)	41 (0.4)	16 (2.7)	39 (2.7)	6 (1.0)	15 (1.9)	24 (5.4)	10 (1.8)	652 (28.2)

**Note**: Unweighted n (weighted %) indicated. Tobacco products included cigarettes, cigars, smokeless tobacco, e-cigarettes, hookah, roll-your-own cigarettes, pipe, snus, bidis, heated tobacco, nicotine pouches and other oral nicotine products.

**a:** Multiple and frequent tobacco product use were assessed in the past 30 days among youth who had used tobacco products in the past 30 days (n = 1977).

Table 2 shows the adjusted Poisson regression model results. The final analytic sample who had information on all study variables was *n* = 17,308. After adjusting for covariates, we found more multiple tobacco product use in the past 30 days among gay/lesbian youth (adjusted Prevalence Ratio (aPR) = 2.35, 95 % CI = 1.15, 4.79) compared to heterosexual/straight youth. Regarding frequent tobacco product use, gay/lesbian (aPR = 2.89, 95 % CI = 1.42, 5.87) and asexual youth (aPR = 2.92, 95 % CI = 1.41, 6.04) had more frequent tobacco product use than heterosexual youth. In sensitivity analyses, negative binomial regression models and zero-inflated Poisson models showed similar findings (**Supplemental Table 1**): Gay/lesbian youth were more likely to report multiple and frequent tobacco product use (aPR range:1.90–2.41), except in the zero-inflated Poisson model for frequent tobacco product use.

**Table 2:** Results of adjusted Poisson regression model on multiple and frequent tobacco use in the past 30 days by sexual identity among total youth respondents in the United States from 2023 National Youth Tobacco Survey (N = 20,503; data was collected in 2023).

	Multiple tobacco product use	Frequent tobacco product use
	Adjusted Prevalence Ratio (95 % CI)	Adjusted Prevalence Ratio (95 % CI)
Sexual identity		
Straight or heterosexual	1.00	1.00
Gay or lesbian	2.35 (1.15, 4.79)	2.89 (1.42, 5.87)
Bisexual, pansexual or queer	1.45 (0.97, 2.17)	1.54 (0.89, 2.68)
Asexual	1.68 (0.97, 2.90)	2.92 (1.41, 6.04)
I am not sure or I am questioning	1.56 (1.02, 2.37)	0.60 (0.18, 2.06)
I don't know what this question means	1.29 (0.67, 2.51)	1.23 (0.47, 3.26)
Something else	1.06 (0.59, 1.89)	1.37 (0.62, 3.01)
Decline to answer	1.03 (0.75, 1.41)	0.88 (0.50, 1.55)

*P*-value 0.025 (0.05/2) was considered statistically significant; Boldface indicates statistical significance.

Adjusted for sex, school level, race, ethnicity, family tobacco use, peer tobacco use, gender identity.

The final analytic sample who had information on all study variables was n = 17,308.

Among youth respondents who reported any tobacco product use in the past 30 days (*n* = 1977), there was no significant difference in the number of tobacco products used across sexual identities. Compared to heterosexual youth, more combustible tobacco product use (e.g., cigarettes, cigars, hookah) was reported by gay/lesbian (aPR = 3.68, 95 % CI = 1.66, 8.12) and asexual youth (aPR = 2.34, 95 % CI = 1.13, 4.83) while more non-combustible tobacco product use (e.g., e-cigarettes, nicotine pouches, smokeless/snus) was reported by bisexual, pansexual, or queer (aPR = 1.44, 95 % CI = 1.02, 2.04) and “questioning” youth (aPR = 1.56, 95 % CI = 1.14, 2.15) (**Supplemental Table 2**).

## 4 Discussion

This study highlights that sexual minority youth (e.g., gay/lesbian, asexual) have more multiple and frequent tobacco product use than heterosexual youth. Our findings align with previous studies showing a greater number of tobacco products used among gay/lesbian youth than heterosexual youth (Creamer et al., 2019; Hart et al., 2020; Tabaac et al., 2021; Azagba and Shan, 2021; Watson et al., 2018). However, this association was attenuated among youth who used any tobacco products in the past 30 days in the current study. These discrepancies may stem from our model adjusting for other factors, unlike the previous study that estimated unadjusted prevalences (Creamer et al., 2019). Alternatively, a cohort effect between pre- and post-COVID data may play a role (Hart et al., 2020; Tabaac et al., 2021). Notably, a greater number of combustible tobacco products were used by gay/lesbian and asexual youths, which might be more harmful than non-combustible use (US Department of Health and Human Services, 2012). Continuous monitoring of multiple tobacco product use among sexual minority youth is needed.

Additionally, our study found more frequent tobacco product use among gay/lesbian and asexual youth compared to heterosexual youth. While frequent use among gay/lesbian youth is well-documented (Azagba and Shan, 2021), our finding regarding asexual youth is novel. Limited research exists on tobacco use among asexual youth. One study observed that there is no significant difference in tobacco use among asexual youth compared to heterosexual youth; however, asexual youth experienced higher tobacco cravings among youth who use tobacco products (Azagba et al., 2025). More research is needed to investigate tobacco use patterns and reasons among asexual youth.

Limitations include possible recall bias, social desirability bias, and selection bias, given the NYTS does not survey youth not attending school. A relatively large group (13.5 %) of students declined to answer the sexual identity question, highlighting challenges in assessing sexual identity in school-based surveys (Spock et al., 2022). Future studies should explore reasons for these declines and employ refined survey assessments. Lastly, we did not examine factors such as substance use or perceived discrimination, which should also be considered.

## 5 Conclusions

In conclusion, this study found greater multiple and frequent tobacco product use among US sexual minority youth compared to heterosexual youth. Findings underscore the importance of developing tailored tobacco prevention strategies and call for action by clinical communities. Clinicians should discuss sexual identity with patients, provide education on tobacco risk, and offer cessation support when needed.

## CRediT authorship contribution statement

**Juhan Lee:** Writing – review & editing, Writing – original draft, Investigation, Formal analysis, Conceptualization. **Rebecca J. Evans-Polce:** Writing – review & editing, Investigation. **Maria A. Parker:** Writing – review & editing, Investigation.

## Declaration of competing interest

The authors declare that they have no known competing financial interests or personal relationships that could have appeared to influence the work reported in this paper.

## Data Availability

Data will be made available on request.

## Appendix A Supplementary data

Supplemental Table 1 Results of adjusted negative binomial and zero inflated Poisson regression models on multiple and frequent tobacco product use in the past 30 days by sexual identity, among overall youth respondents (N=20,503). Image 1

Supplemental Table 2 Results of adjusted Poisson regression models on the three past 30 day supplemental outcomes by sexual identity. Image 2

## References

[bb0005] Azagba S., Shan L. (2021). Disparities in the frequency of tobacco products use by sexual identity status. Addict. Behav..

[bb0010] Azagba S., Ebling T., de Silva G.S.R. (2025). Disparities in tobacco use and cravings among sexual and gender minority adolescents in the United States. Prev. Med. Rep..

[bb0015] Bates N., Chin M., Becker T. (2022).

[bb0020] Birdsey J., Cornelius M., Jamal A. (2023). Tobacco Product Use Among U.S. Middle and High School Students — National Youth Tobacco Survey, 2023. MMWR Morb. Mortal Wkly. Rep..

[bb0025] Blosnich J., Lee J.G.L., Horn K. (2013). A systematic review of the aetiology of tobacco disparities for sexual minorities. Tob. Control..

[bb0030] Centers for Disease Control and Prevention (CDC) (2020). National Youth Tobacco Survey (NYTS). https://www.cdc.gov/tobacco/data_statistics/surveys/nyts/index.htm.

[bb0035] Creamer M.R., Sherry, Jones E., Gentzke A.S., Jamal A., King B.A. (2019). Tobacco product use among high school students — youth risk behavior survey. United States.

[bb0040] Hart J.L., Ridner S.L., Wood L.A. (2020). Associations between tobacco use patterns and demographic characteristics of sexual minority and heterosexual youth: results from a nationwide online survey. Tob. Prev. Cessat..

[bb0045] Jamal A., Park-Lee E., Birdsey J. (2023). Morbidity and Mortality Weekly Report Tobacco Product Use Among Middle and High School Students-National Youth Tobacco Survey. United States, 2024.

[bb0050] Meyer I.H. (2003). Prejudice, Social stress, and mental health in lesbian, gay, and bisexual populations: conceptual issues and research evidence. Psychol. Bull..

[bb0055] Eaton D.L., Kwan L.Y., Kathleen Stratton (2018). Public health consequences of E-cigarettes.

[bb0060] Park-Lee E., Jamal A., Cowan H. (2024). Notes from the field: E-cigarette and nicotine pouch use among middle and high school students — United States, 2024. MMWR Morb. Mortal Wkly. Rep..

[bb0065] Robichaud M.O., Seidenberg A.B., Byron M.J. (2020). Tobacco companies introduce “tobacco-free” nicotine pouches. Tob. Control..

[bb0070] Spock A., Popkin R., Barnhart C. (2022). Strategies to improve measurement of sexual orientation and gender identity among youth. J. Adolesc. Health.

[bb0075] Tabaac A.R., Charlton B.M., Tan A.S.L., Cobb C.O., Sutter M.E. (2021). Differences in tobacco product use by sexual orientation and violence factors among United States youth. J. Pediatr..

[bb0080] UCLA Williams Institute (2025). Nonbinary LGBTQ Adults in the United States.

[bb0085] United States Department of Health and Human Services (2012).

[bb0090] US Department of Health and Human Services (2012). Preventing Tobacco Use Among Youth and Young Adults. https://www.ncbi.nlm.nih.gov/books/NBK99237/pdf/Bookshelf_NBK99237.pdf.

[bb0095] Wang T.W., Gentzke A.S., Creamer M.R. (2019). Tobacco product use and associated factors among middle and high school students — United States, 2019. MMWR Surveill. Summ..

[bb0100] Watson R.J., Lewis N.M., Fish J.N., Goodenow C. (2018). Sexual minority youth continue to smoke cigarettes earlier and more often than heterosexuals: findings from population-based data. Drug Alcohol Depend..

