# Supplementary material for: Multiple and frequent tobacco product use by sexual minority youth in the United States: Results from the 2023 National Youth Tobacco Survey
Source: Prev Med Rep. 2025 Apr 10;54:103069. doi: 10.1016/j.pmedr.2025.103069 (PMC12020873; doi:10.1016/j.pmedr.2025.103069)
Supplement: Supplemental Table 1 — Results of adjusted negative binomial and zero inflated Poisson regression models on multiple and frequent tobacco product use in the past 30 days by sexual identity, among overall youth respondents (N=20,503). [file mmc1.pdf]

EINSTEIN
Albert Einstein College of Medicine
Office of Human Research Affairs
Montefiore

Science at the heart of medicine

Institutional Review Board

Albert Einstein College of Medicine
FWA #00023382

Montefiore Medical Center
FWA #00002558

North Bronx Healthcare Network
FWA #00009807

Yeshiva University
FWA #00000140

White Plains Hospital
FWA #00023382

East Campus IRB
Jack and Pearl Resnick Campus
1300 Morris Park Ave., Belfer 1002
Bronx, NY 10461
718.430.2237 fax 718.430.8817

West Campus IRB
Montefiore Medical Center
3308 Rochambeau Avenue
Bronx, NY 10467
718.798.0406 fax 718.798.5657

Notification of Determination

Date: January 23, 2025

Principal Investigator: Juhan Lee

**Study Title:**	Understanding tobacco and substance use among youth and adults at the population level: Secondary data analyses of publicly available, de-identified US national datasets		
**IRB #:**	2024-16347	**Reference #:**	119541
**Type of Submission:**	Submission Response for Initial Review Submission form		
**Determination Date:**	01/23/2025	**Expiration Date:**	01/22/2028

This is to inform you that the above named application has been reviewed by the Einstein IRB. In accordance with 45 CFR 46.102(f), this study does not meet the definition of human subjects research, as the investigators are not interacting or intervening with living individuals or their identifiable information.

Note: It is the PI's responsibility to obtain any additional local institutional or departmental required approvals prior to initiating this study.

Re-review by the IRB will be required if any substantive change is made in the protocol during the course of the study, to determine whether or not the study still qualifies as "not human subjects".

Data Use Agreements: If you are releasing data to an external site/entity/collaborator, you are required to obtain a DUA (Data Use Agreement). This may be obtained through the Research Agreement Request Portal (https://einsteinmed.co1.qualtrics.com/jfe/form/SV_8fgVaus0Bpcpeux).

Expiration Notice: Institutional approval for this study is limited to the period specified above. In order to gain re-approval, you must submit a Progress Report by 12/22/2027. To facilitate this, iRIS will send an email reminder 60 days prior to the due date. When this project is completed, submit a final Progress Report to close the file.

Reviewed Documents: To obtain a list of documents that were reviewed with this submission, follow these steps: Go to My Studies and open the study – Click on Submissions History – Go to Completed Submissions – Locate this submission and click on the Details button to view a list of submitted documents and their outcomes.
