# Supplementary material for: Multiple and frequent tobacco product use by sexual minority youth in the United States: Results from the 2023 National Youth Tobacco Survey
Source: Prev Med Rep. 2025 Apr 10;54:103069. doi: 10.1016/j.pmedr.2025.103069 (PMC12020873; doi:10.1016/j.pmedr.2025.103069)
Supplement: Supplemental Table 2 — Results of adjusted Poisson regression models on the three past 30 day supplemental outcomes by sexual identity. [file mmc2.docx]

**Supplemental Table 1**. Results of adjusted negative binomial and zero inflated Poisson regression models on multiple and frequent tobacco product use in the past 30 days by sexual identity, among overall youth respondents (N=20,503)

|  | Negative binomial | | Zero-inflated Poisson | |
| --- | --- | --- | --- | --- |
|  | Multiple tobacco product use | Frequent tobacco product | Multiple tobacco product use | Frequent tobacco product |
|  | Adjusted Prevalence Ratio (95% CI) | Adjusted Prevalence Ratio (95% CI) | Adjusted Prevalence Ratio (95% CI) | Adjusted Prevalence Ratio (95% CI) |
| Sexual identity |  |  |  |  |
| Straight or heterosexual | 1.00 | 1.00 | 1.00 | 1.00 |
| Gay or lesbian | 2.03 (1.16, 3.55) | 2.41 (1.42, 4.09) | 1.90 (1.14, 3.17) | 1.60 (0.80, 3.20) |
| Bisexual, pansexual or queer | 1.51 (0.98, 2.33) | 1.82 (1.03, 3.21) | 1.19 (0.79, 1.81) | 1.55 (0.96, 2.49) |
| Asexual | 1.27 (0.77, 2.11) | 2.25 (1.11, 4.56) | 1.09 (0.67, 1.79) | 2.23 (1.12, 4.45) |
| I am not sure or I am questioning | 1.10 (0.73, 1.66) | 0.69 (0.25, 1.88) | 0.81 (0.45, 1.46) | 0.75 (0.29, 1.98) |
| I don’t know what this question means | 0.95 (0.47, 1.93) | 0.58 (0.35, 0.97) | 0.99 (0.68, 1.44) | 0.75 (0.47, 1.22) |
| Something else | 0.93 (0.54, 1.60) | 1.10 (0.53, 2.27) | 0.92 (0.56, 1.51) | 0.99 (0.60, 1.63) |
| Decline to answer | 0.99 (0.73, 1.33) | 0.93 (0.51, 1.72) | 1.04 (0.78, 1.39) | 0.96 (0.50, 1.86) |

P-value 0.025 (0.05/2) was considered statistically significant; Boldface indicates statistical significance

Adjusted for sex, school level, race, ethnicity, family tobacco use, peer tobacco use, gender identity.

**Supplemental Table 2**. Results of adjusted Poisson regression models on the three past 30 day supplemental outcomes by sexual identity

|  | Multiple tobacco product use among youth who had any tobacco use (n=1,977) | The number of combustible tobacco products used among overall respondents (N=20,503) | The number of non-combustible tobacco products used among overall respondents (N=20,503) |
| --- | --- | --- | --- |
|  | aPR (95% CI) | aPR (95% CI) | aPR (95% CI) |
| Sexual identity |  |  |  |
| Straight or heterosexual | 1.00 | 1.00 | 1.00 |
| Gay or lesbian | 1.39 (0.99, 1.95) | 3.68 (1.66, 8.12) | 1.82 (0.95, 3.49) |
| Bisexual, pansexual or queer | 0.99 (0.75, 1.29) | 1.74 (0.92, 3.31) | 1.44 (1.02, 2.04) |
| Asexual | 1.07 (0.70, 1.64) | 2.34 (1.13, 4.83) | 1.46 (0.87, 2.44) |
| I am not sure or I am questioning | 0.93 (0.55, 1.58) | 1.58 (0.71, 3.51) | 1.56 (1.14, 2.15) |
| I don’t know what this question means | 1.02 (0.75, 1.39) | 1.03 (0.48, 2.19) | 1.40 (0.70, 2.80) |
| Something else | 0.95 (0.64, 1.40) | 1.23 (0.54, 2.81) | 1.00 (0.57, 1.76) |
| Decline to answer | 1.03 (0.88, 1.20) | 0.82 (0.54, 1.24) | 1.14 (0.81, 1.60) |

aPR= adjusted prevalence ratio; Boldface indicates statistical significance (p<0.05)

Adjusted for sex, school level, race, ethnicity, family tobacco use, peer tobacco use, gender identity

“combustible tobacco products” included cigarettes, cigars, hookah, roll-your-own cigarettes, pipe, bidis

“non-combustible tobacco products” included smokeless/snus, e-cigarettes, heated tobacco, oral nicotine products, nicotine pouches.
